# Special Issue “Fungal Burden in Different Countries”

**DOI:** 10.3390/jof4030080

**Published:** 2018-07-03

**Authors:** Malcolm D. Richardson, Donald C. Cole

**Affiliations:** 1 Mycology Reference Centre Manchester, Manchester University NHS Foundation Trust, and Division of Infection, Immunity and Respiratory Medicine, University of Manchester, Manchester, UK; 2 Dalla Lana School of Public Health, University of Toronto, Toronto, Canada

Adults and children living in many countries face a combined burden of infectious diseases including fungal infections (for example, tinea capitis, recurrent vulvo-vaginal thrush, chronic pulmonary aspergillosis, candidemia). Focused surveys have estimated the burden of specific diseases, such as HIV and malaria but little data exists for fungal infection. Estimates of the burden of disease in adults in many countries largely rely on modeling using sparse data. Mapping of the global burden of fungal disease at a country level has shown marked diversity of disease across many continents. Geographical mapping is useful to understand patient exposure in defined ecological niches. Fungi are distributed in a plethora of ecosystems and each year new reservoirs are discovered, which helps researchers and clinicians to better understand the epidemiology and burden of fungal disease. The available published literature describing the burden of disease in adults in many areas reports high amounts of uncertainty in the estimates, mainly as a result of weak or absent disease surveillance combined with the absence of vital registration in these regions. Furthermore, few published studies have used hospital-based data, and those that have done so have several limitations: they rarely use universally accepted coding systems and case definitions for the illnesses described; and data capture is seldom complete because of various logistical challenges in the use of electronic medical systems.

Insufficient data about fungal disease burden have been acknowledged as a major barrier to public health decision making by global authorities. The Global Action Fund for Fungal Infections (GAFFI), a foundation based in Switzerland has grasped the challenge of the paucity of knowledge regarding these diseases. Various collections of country burden estimates have been published over the past few years. A discussion paper outlining what is known so far and the accuracy of the data has been published recently in this journal [1]. To set the scene, fungal diseases kill more than 1.5 million and affect over a billion people. Serious fungal infections occur as a consequence of other health problems including asthma, AIDS, cancer, organ transplantation, and corticosteroid therapies. Early accurate diagnosis allows prompt antifungal therapy. However, this is often delayed or unavailable leading to death, serious chronic illness or blindness. Recent global estimates have found 3,000,000 cases of chronic pulmonary aspergillosis, ~223,100 cases of cryptococcal meningitis complicating HIV/AIDS, ~700,000 cases of invasive candidiasis, ~500,000 cases of *Pneumocystis jirovecii* pneumonia, ~250,000 cases of invasive aspergillosis, ~100,000 cases of disseminated histoplasmosis, over 10,000,000 cases of fungal asthma, and ~1,000,000 cases of fungal keratitis occur annually.

In the latest collation of studies published in the Journal of Fungi (http://www.mdpi.com/journal/jof/special_issues/fungal_burden) from South American (Argentina, Colombia, Uruguay), Africa (Burkina Faso, Cameroon, Malawi, Mozambique), the Central Asia and South-East Asia (Jordan, Kazakhstan, Malaysia), Scandinavia (Norway), and Eastern Europe (Romania, Serbia) the authors have endeavored to link, where possible clinical and demographic surveillance to comprehensively describe the burden of disease in their country. The spectrum of papers from diverse regions of the world has shown how fungal disease burden data can be obtained through the integration of clinical surveillance data into existing investments in demographic surveillance systems. These systems are by no means universal. In settings where clinicians have access to high-quality clinical, laboratory, and imaging investigations, physician-coded hospital data are superior to verbal autopsy data. This is apparent in the collection of country burdens published here. Many fungal diseases are not notifiable and definitive diagnosis is lacking or nonexistent. It is quite clear that the estimates presented are probably a substantial under-representation of the true burden of fungal disease in many countries.

A crucial component of the ability of individual countries to produce data about the prevalence of fungal disease is the provision of appropriate and accurate diagnostic tests. The current status of mycology diagnostic services in low- and middle-income countries is virtually unknown. Where such surveys have been conducted (for example, the UK and a number of Asian countries) the data can be used to provide guidance to policy makers to increase provision of mycology reference laboratories. In turn, these centres can improve notification of fungal disease and estimates of fungal burdens. Access to a national (or regional) specialized mycology laboratory undertaking disease surveillance and registry is important. It is hoped that the ongoing mapping and reporting of fungal disease across the globe will support greater efforts by countries and health organisations to implement essential diagnostic tests in order to improve the accuracy of prevalence estimates in regions where these do not exist, to implement better treatment and to reduce the burden of chronic and systemic fungal disease. The investigation of fungal disease patterns in diverse settings across the globe should be used to guide public health policy and stimulate appropriate research. 
